# Re-evaluating vocal production learning in non-oscine birds

**DOI:** 10.1098/rstb.2020.0249

**Published:** 2021-10-25

**Authors:** Carel ten Cate

**Affiliations:** Institute of Biology, Leiden University, PO Box 9505, 2300 RA Leiden, The Netherlands

**Keywords:** vocal learning, songbirds, parrots, non-songbirds, phylogeny, learning mechanisms

## Abstract

The study of vocal production learning in birds is heavily biased towards oscine songbirds, making the songbird model the reference for comparative studies. However, as vocal learning was probably ancestral in songbirds, interspecific variations might all be variations on a single theme and need not be representative of the nature and characteristics of vocal learning in other bird groups. To assess the possible mechanisms of vocal learning and its evolution therefore requires knowledge about independently evolved incidences of vocal learning. This review examines the presence and nature of vocal production learning in non-songbirds. Using a broad definition of vocal learning and a comparative phylogenetic framework, I evaluate the evidence for vocal learning and its characteristics in non-oscine birds, including well-known vocal learners such as parrots and hummingbirds but also (putative) cases from other taxa. Despite the sometimes limited evidence, it is clear that vocal learning occurs in a range of different, non-related, taxa and can be caused by a variety of mechanisms. It is more widespread than often realized, calling for more systematic studies. Examining this variation may provide a window onto the evolution of vocal learning and increase the value of comparative research for understanding vocal learning in humans.

This article is part of the theme issue ‘Vocal learning in animals and humans’.

## 1. Introduction

Learning to produce particular sounds, i.e. vocal production learning, is a crucial feature for the development of human language. It is also a feature that is, at best, marginally present in our nearest relatives, the great apes and other primate species [1]. Nevertheless, evidence for vocal learning is present for some other mammalian groups, such as several dolphins and whales (e.g. [2,3]), some bat species (e.g. [4]), harbour seals (e.g. [5]) and Asian elephants [6]. However, well before the evidence for vocal learning in non-human mammals emerged, it was already known that several bird species require exposure to a model to sing normal songs (e.g. [7]) or are capable of mimicking other sounds, such as human speech. More specifically, this knowledge concerned vocal learning in oscine songbirds (hereafter referred to as ‘songbirds’) and parrots, which both have a rich history of being bred or raised by aviculturists. It is thus no wonder that when scientists developed a greater interest in vocal learning they used these groups, and in particular songbirds, for more systematic investigations. This confirmed the widespread presence of vocal learning in songbirds and revealed many features of the processes involved and their neural and genetic underpinnings. It gave rise to the classical model for the song learning process, primarily based on the pioneering studies by Marler (e.g. [8]) on the white-crowned sparrow, demonstrating a two-phase learning process. During a sensory learning phase in the first months of life, juvenile white-crowned sparrows memorize a song. In the next spring, during the so-called sensory-motor phase, the memorized song serves as the template to which a gradually emerging subsong is being shaped towards a crystallized version by auditory self-feedback. Over the years, when more and more species were examined, it became apparent that songbird species vary a lot in the characteristics and details of the learning process, such as the timing of the sensory phase, the required experience, the constraints on the process, the impact of social factors, etc. (e.g. [9,10]).

The abundance of songbird species (roughly half of the approximately 10 000 bird species), their diversity in song and song learning characteristics, and the ease with which several species can be bred and raised in captivity has made this the best-studied clade for various aspects of vocal production learning. However, as vocal learning may already have been present in the common ancestor of all songbirds, all variations among songbirds might be considered variations on a single theme. Therefore, if we want to understand how and why vocal production learning evolved, the behavioural and neural mechanisms underlying it, and how it compares to vocal learning in mammals, including humans, we need to examine its manifestation in other groups in which it evolved independently and compare the characteristics of the learning process among these groups. There is, however, a noticeable contrast between our detailed knowledge about songbird song learning and knowledge on the presence and nature of vocal learning in other bird groups. Despite the fact that vocal learning is known from parrots and hummingbirds [11,12], knowledge about the learning processes involved is still limited. For other bird groups, even less knowledge about (putative) vocal learning is available. There may be several reasons for this. The vocalizations in many groups lack more complex structures or show no obvious interindividual variations in element types and sequences. Early studies on some representatives of those groups showed that birds deafened at an early age (chickens—[13]; ringdoves—[14]) or cross-fostered to another species (*Streptopelia* doves—[15]) still developed seemingly normal species-specific sounds, which may have discouraged further exploration. However, these studies concerned only a few species and limited numbers of individuals and no detailed quantitative analyses, so more subtle modifications by some form of learning might have gone unnoticed. Also, several studies on suboscine flycatchers, sister group to the songbirds, initially indicated no evidence of learning (see below), supporting the idea of vocal learning being restricted to a few taxa. However, over the years, there have been scattered reports on species other than songbirds, parrots or hummingbirds that indicated the presence of some form of vocal learning in individuals raised with other species or under specific conditions.

The aim of this review is to assess what is known about vocal learning in non-songbirds: Where can it be found? What is learned in these species? What is the nature of the learning processes involved? To qualify a phenomenon as vocal learning I use the definition by Janik & Slater [16]): (Vocal) ‘signals that are modified in form as a result of experience with those of other individuals’. However, I leave out that learning needs to result from experience with the *vocalizations* of other individuals, thus including other types of experience with other individuals that may affect vocalizations. This definition is broad and not restricted to any specific mechanism, time frame or life stage. It also includes modifications of existing sounds. Important, however, remains another qualification of the Janik & Slater definition, which is that ‘the experience can lead to signals that are either similar or dissimilar to the model’. In other words, the direction of vocal changes as a result of experience needs to show a relation to an identifiable model sound. This excludes vocal changes such as those occurring in noisy environments for which it is also often not (yet) clear whether any vocal learning is involved or whether such changes are more ‘hard-wired’ like, for instance, the Lombard effect.

**Figure 1.**
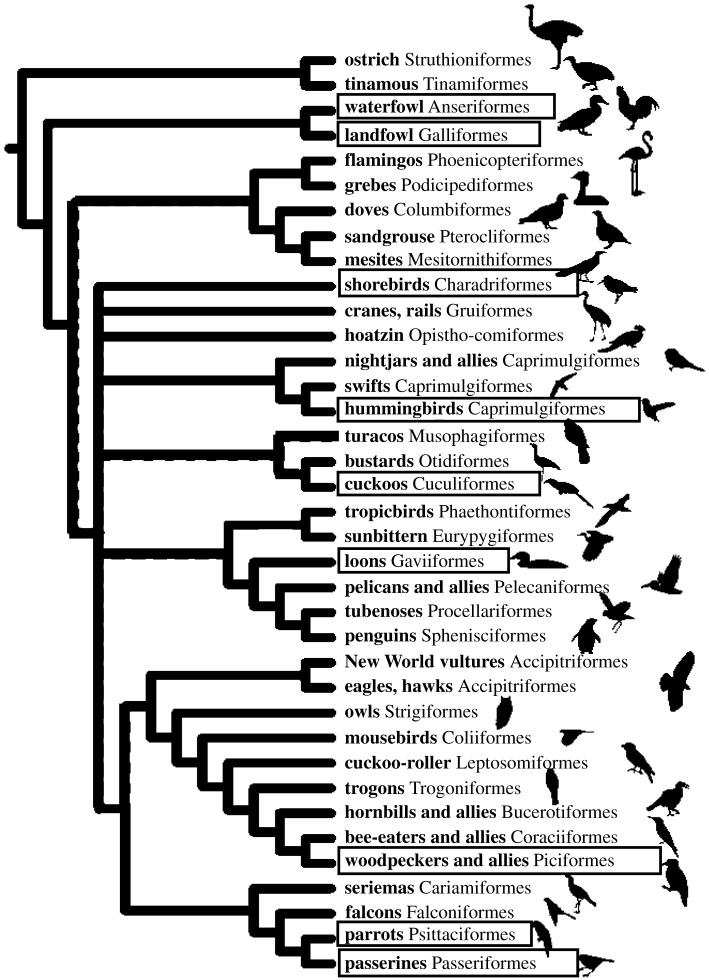
Consensus tree depicting the phylogenetic relationship between the different bird orders. Boxes indicate the orders discussed in this paper. Adapted from Reddy *et al*. [17] with permission from the Oxford University Press.

Below I review the evidence for vocal production learning in different orders of non-songbirds, arranged according to their increasing phylogenetic distance from songbirds (figure 1). Doing so reveals that ‘vocal production learning’ is a mixed bag of processes that may show both similarities and differences to the songbird system. Thereafter, I discuss what can be learned from the distribution of vocal learning among bird groups, characterize the different forms of learning observed and discuss what the findings imply about the evolution of vocal learning.

### (a) Suboscines

**Figure 2.**
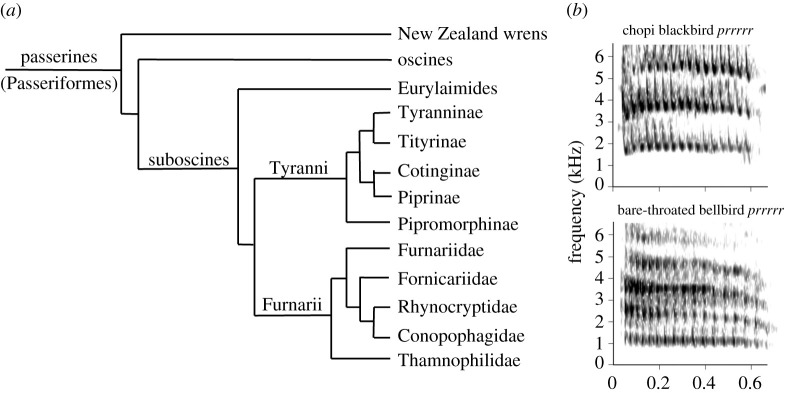
(*a*) Phylogenetic relationships within the passerines, with a focus on the New World suboscines (Tyranni and Furnarii) (based on de Lima *et al*. [18]). (*b*) Incidence of allospecific song copying by a bare-throated bellbird (Cotinginae) imitating a chopi blackbird (from Kroodsma *et al*. [19] with permission from the *Wilson Journal of Ornithology*).

The order of Passeriformes contains three suborders (figure 2*a*): the basal clade of New Zealand wrens, the oscine songbirds, and the suboscines. No studies have been done on the four species of New Zealand wrens, but several studies have examined the presence of vocal learning in suboscines. The suboscines contain about 1400 species (species numbers here and elsewhere are based on [20]), divided between the Eurylaimides (Old World suboscines—71 species) and the New World suboscines (1331 species). There are no reports of vocal learning in Old World suboscines, but they have not been very well examined. Experimental studies on several New World suboscines have demonstrated the absence of vocal learning. Spotted antbirds, belonging to the Thamnophilidae, reared in acoustic isolation or with exposure to heterospecific song, developed normal songs [21]. Also, several tyrannid flycatchers that were hand reared and acoustically isolated, deafened or exposed to heterospecific songs developed normal conspecific songs (Eastern phoebe—[22]; willow flycatcher, alder flycatcher—[23]). However, strong evidence for vocal learning is present in two species of bellbirds, belonging to the Cotinginae, a group well embedded within the Tyranni. Three-wattled bellbirds (*Procnias tricarunculata*) show different vocal dialects. Detailed field observations demonstrated that these dialects can change over the years [19]. Such changes are observed in individual males, while other males may combine different dialects. Clear evidence for vocal imitation is shown by a bare-throated bellbird (*Procnia nudicollis*), caged as a juvenile with a female chopi blackbird (*Gnorimopsar chopi*). It copied two different blackbird sounds (e.g. figure 2*b*), while also producing a deviant bare-throated bellbird vocalization [19]. When housed with conspecifics later on this bird maintained the deviating bellbird vocalization and did not copy normal bellbird vocalizations. This suggests the learning occurred during an early sensitive phase, influenced by social interactions with the heterospecific tutor—a pattern similar to that known for several songbird species. For other species within the family Cotinginae, there are no reports of vocal learning although there is some evidence that male long-tailed manakins (*Chiroxiphia linearis*), in which pairs of males display cooperatively, over time adjust some details of their rather simple species-specific song to match those of their partner [24]. This could indicate some plasticity in what for the rest seems to be a developmentally rather fixed song development. The observations on the bellbirds strongly suggest an independent evolution of vocal learning within the genus *Procnia*, unless one assumes a series of losses in other branches of the suboscines and among other Cotinginae. To date, nothing is known about the neural substrate underlying this vocal learning. However, the eastern phoebe, a tyrannid [22], and also the scale-backed antbird (*Willisornis poecilinotus*) belonging to the Thamnophilidae, show no indications of vocal learning but have a specialized forebrain area that is homologous to the oscine song nucleus RA (robust nucleus of arcopallium) in several neural characteristics [18,25]. Whether this similarity extends to the Cotinginae and resembles an ancestral situation, providing the neural foundation for the independent evolution of vocal learning in oscines and suboscines, awaits further exploration.

### (b) Parrots

**Figure 3.**
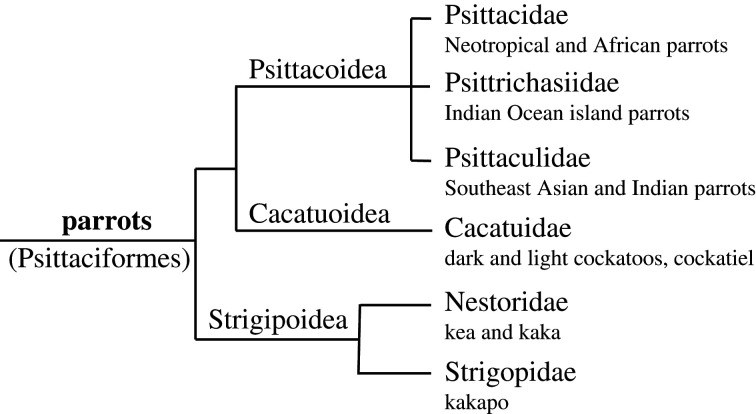
Parrot phylogeny, based on Joseph *et al*. [26]. Extensive vocal learning abilities are known from species belonging to the three families of Psittacoidae and for the Cacatuoidae. The Strigipoidae show some dialectal variation, indicating limited vocal learning only.

The parrot order (Psittaciformes) consists of 414 species [20], grouped into three ‘superfamilies’ incorporating six families (figure 3). Just like many songbirds, parrots use vocalizations to defend breeding territories, but many species also forage in groups in which various calls have an important role in the social dynamics of group living [27].

Geographic variation of call notes is present in a broad range of species belonging to the Psittacidae, Psittaculidae, Cacatuidae and Strigopidae, i.e. seems present in all families [28]. A clear dissociation between vocal and genetic variation, as observed in Australian ringneck parrots [29] and yellow-naped amazons [30] suggests that such call dialects result from learning. Call learning is also demonstrated by a field study showing that individual contact calls of cross-fostered green-rumped parrotlet (*Forpus passerinus*) nestlings show higher similarity to contact calls of the (foster) parents than to calls of other individuals [31]. Once acquired, parrot calls may also change in the context of social interactions. Extensive laboratory studies, for instance, have shown that group-housed budgerigars converge in their contact calls (e.g. [32,33]). Also, male budgerigars may change a call to become similar to their female's call [34].

The call changes in adult parrotlets and budgerigars may take some time to develop. However, in some parrot species, call changes can occur on a much shorter time scale, over the course of an interaction. For instance, the contact calls of orange-fronted conures (*Aratinga canicularis*) can either converge or diverge to playbacks of calls [35]. Such rapid call convergence and divergence has also been observed in other parrot species, although there is variation in both the degree and direction of the resulting changes, possibly depending on the nature of the social dynamics of the species and individuals concerned [36].

However, the clearest and most familiar evidence for vocal learning in parrots comes from parrots kept as pets, mimicking human speech or other sounds from their environment. For instance, two budgerigars, Puck and Disco, made headlines in the popular press because of their extensive repertoire of speech imitations [37] and many impressive examples of such copying in several other species can be found in YouTube movies or on various websites (e.g. https://en.wikipedia.org/wiki/Talking_bird#cite_note-PetMD-9). Such examples of vocal production learning of sounds well outside the species-specific range are known for the three families of the Psittacoidea and for the Cacatuidea, but not for the Strigipoidea. A well-documented example of allospecific vocal imitation in the Cacatuidea concerns field observations on two galahs (*Cacatua roseicapilla*) accidentally raised by Major Mitchell's cockatoos (*Cacatua leadbeateri*). Most calls in their repertoire were copied from the Major Mitchell's cockatoos, although some others were galah calls [38], indicating that the learning is call-type dependent. The copying of Major Mitchell's calls must have occurred early in life, influenced by experience with parents.

Based on the variation in the ways and extent in which parrot vocalizations can be affected by experience, Bradbury & Balsby [27] suggested the presence of three types of learning, primarily based on the context of learning rather than the mechanisms that might be involved: early acquisition of contact calls, vocal modifications related to social affiliations in adult birds, and immediate vocal convergence and divergence during a call interaction. Different mechanisms may underly these types of vocal modifications. The learning shown by the galahs raised by the Major Mitchell's cockatoos, as well as the learning of contact calls from exposure at an early age, may be comparable to song learning in songbirds. It originates from exposure to a vocalization in a social context and suggests the presence of a memorization phase preceding a sensorimotor phase as occurs in songbirds. Call convergence, such as observed in groups of adult budgerigars, might be due to a second, incremental, type of learning process resembling operant conditioning. Differential responses to different variants of an individual's call might reinforce gradual and directional call modifications. This has been demonstrated in experiments in which budgerigars modified their contact call in a specific direction by being selectively reinforced with a food reward for calls approaching the required target [39]. Budgerigars could also be trained to produce calls that diverged from the target and even to produce two distinct call variants [39]. The targets were not played to the birds, so the changes are due to shaping by selective reinforcement. However, a later experiment [40], in which the target sound was also played to the budgerigars, resulted in a more rapid acquisition of matching calls, indicating that the presence of a model enhanced the learning.

A different process may underly the short-term call changes over the course of an interaction or in response to a playback, in which vocalizations converge or diverge from one a parrot hears at that moment. Whether such modifications should be considered a form of vocal production learning is arguable. It may be an automatic, short-term, process leaving no clear identifiable trace after the interaction. Such changes resemble ‘vocal accommodation’—a process described for humans and several primate species, in which vocal features of two interacting individuals become more alike or diverge over the course of an interaction [41]. It need not involve memory formation, usage of a stored template or reinforcement learning.

How imitations of human speech and other sounds relate to the above-mentioned processes and whether these sounds are generated using a memorized template of such sounds is unclear. The famous grey parrot Alex, for instance, acquired his repertoire of human words not just by imitation of sounds modelled for him, but also by selective reinforcement shaping the production of desired sounds [42]. This suggests that vocal imitations might originate from a combination of processes, as is also suggested by the budgerigar experiments in Manabe *et al*. [41].

So, vocal learning in parrots might result from (a combination of) different types of learning processes, rather than a single one. What is not clear at present is how the above types of vocal modifications are distributed over various parrot taxa. Also not clear is whether they are really separate processes (e.g. one being songbird-like template learning, another one being a form of operant conditioning), or different variants of what is in principle the same process (similar to variations in song learning characteristics in songbirds being variations on a single theme). Nevertheless, differentiation among various types of learning is also suggested by variation in the neural structure of the vocal system in parrots. This consists of a so-called ‘core’ region, in which gene expression and neural connectivity are comparable to the song systems of songbirds, and a ‘shell’ region which is specific to parrots [43]. Chakraborty *et al*. [43] showed considerable differences in relative size between core and shell regions among parrot species and suggest these could reflect functional differences, with species considered to have more complex communication or learning abilities having larger and more conspicuous shell nuclei relative to the core nuclei compared to species having more limited abilities. However, the precise links between these areas and different aspects of learning still awaits further investigation. The shell region is only present in a rudimentary form in the kea (*Nestor notabilis*). The kea belongs to the Strigopoidea (figure 2*a*), the earliest divergent branch from the parrot phylogenetic tree. There are no reports of mimicking of other species or other sounds from this group. However, similar to many other parrot species, keas show local dialects in social calls [44]. So, to end this section with a note on the evolution of parrot vocal learning: the mechanisms underlying call modification as a result of social interactions seem most widespread and might reflect the ancestral state, relying on the core region, with the more extensive vocal mimicking abilities of novel (allospecific or other) sounds as present among the Psittacoidea and Cacatuoidea evolving later on, requiring more extensive, more long term, or more specialized mechanisms of memorization and control, and might rely more on the shell region.

### (c) Woodpeckers and allies

Toucans (Ramphastidae, 50 species—[20]) are a family within the Piciformes (woodpeckers and allies). Wagner [45, p. 73] classified emerald toucanets (*Aulacorhynchus prasinus*) as ‘good mimics’, based on the following field observations:

The calls of the Emerald Toucanet are so varied that is it impossible to describe them in detail. One thing they have in common, they are all loud and penetrating. These birds are good mimics, but they apparently imitate a given call only so long as they continue to hear the original caller from time to time. During the season in the high mountains I heard most frequently the yow yow call of the Mexican trogon (Trogon mexicanus), the rayg rayg of the quetzal (Pharomachrus micinno), or the eeya eeya of the Azure-hooded jays (Cyanolyca mitrata). In the non-breeding range, I heard none of these calls, but instead the dir-rit of the common Jalapa trogon (Trogon collaris) or the typical hoot hoot of the male Lesson's motmot (Momotus momota lessonii). Sometimes these mimicked calls deceived me, and I would expect to see a different bird in the foliage.

However, since then there have been no studies confirming these observations. Recordings of this species available at the website Xeno-canto (https://www.xeno-canto.org/) are all very similar, consisting of a repetition of a monosyllabic sound with no indication or mentioning that they might copy any other species. The striking contrast between Wagner's firm statements and current evidence leaves the incidence of vocal learning in this species unresolved. Also, various toucan species are frequently kept as pets but none of the online platforms on which owners exchange information mentions any indication of vocal learning.

### (d) Loons

Within the small order of Gaviiformes (5 species—[20]), a study on common loons (*Gavia immer*) showed within-individual vocal modifications in yodel calls [46]. Loon yodels have a characteristic species-specific structure that is common to all individuals, but different individuals differ in spectrotemporal parameters of the introductory notes. The characteristics of yodels remain relatively stable between years for loons that keep the same territory. However, when loons change territory, moving to a different lake between years, the characteristics of the yodel change. Of course, it could be that a territory change also involves other changes, e.g. physiological ones, that may affect the sound characteristics. However, the striking finding was that nine birds that took over another loon's territory all changed their yodel, making it more distinct from the yodel that was produced by the previous territory owner [46].

What drives this directional change is unknown. It might be that the previous owner's yodel served as a model for differentiating, like the call divergence observed in some parrot species. However, there are also indications that resident loons differentiate in their responses to familiar birds and to strangers, which might make the responses of other individuals a factor driving the changes [46]. Whether such experience-based vocal modifications can be found in other loon species is unknown.

### (e) Cuckoos

**Figure 4.**
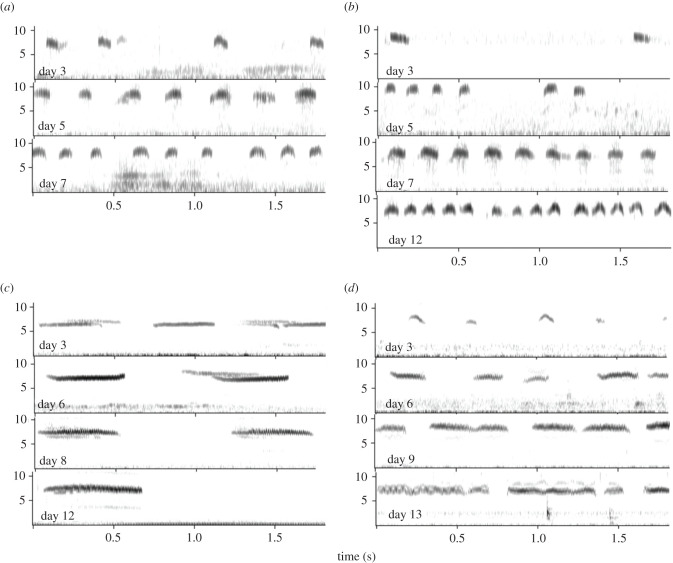
When raised by its usual host (superb fairy-wren) the begging calls of Horsfield's bronze cuckoo chicks (*b*) are similar to those of fairy-wren chicks (*a*). However, when raised by a novel host, the buff-rumped thornbill, the cuckoo begging calls (*d*) become similar to those of thornbill chicks (*c*), despite the absence of host chicks. (From [48] with permission from the John Wiley & Sons).

Cuckoo species (Cuculiformes—151 species—[20]) are avian brood parasites that lay their eggs in the nest of a host species. After hatching, young of various species may evict the host eggs or young and hence are alone in the nest. Cuckoo chicks show several adaptations to induce the hosts to feed them, among which is a similarity between the begging calls of the chicks and those of host chicks (e.g. [47]). Such similarity might result from evolutionary selection on cuckoo begging calls in species specializing on a particular host. However, in some species, there is developmental plasticity in the begging call structure with chicks showing changes making their begging calls more similar to the host begging calls. A clear example is the Horsfield's bronze cuckoo (*Chalcites basalis*—[48]). The usual host for this species is the superb fairy-wren (*Malurus cyaneus*) and newly hatched cuckoo young produce begging calls similar to those of the wrens. However, if such young are transferred to another host species, the buff-rumped thornbill (*Acanthiza reguloides*), they modify their begging calls in frequency bandwidth and duration over the nestling period to become highly similar to those of nestling thornbills (figure 4).

As a cuckoo chick is raised in a nest without any host chicks, vocal similarity to host chicks cannot be obtained by copying. Langmore *et al*. [48] describe how, after transfer to the new host species, cuckoo chicks first increase the variability in their begging calls but next develop a call similar to that of the new host. This call change is most likely due to host parents responding with more feeding after a chick gives a begging call resembling that of conspecific chicks. So a parental perceptual bias tuned to particular begging call characteristics shapes the cuckoo calls to become like those of the host species, ending up outside the range of their initial begging calls. Such experience-based call modification is also known from the common cuckoo (*Cuculus canorus*) [49]. Playbacks of different begging call types to hosts resulted in different parental feeding rates, thus confirming that host behaviour can provide the differential reinforcement to drive chick call modifications. However, another cross fostering study on a specialist cuckoo species, the great spotted cuckoo (*Clamator glandarius*), showed some host-related variation in a number of notes per call, but no increased similarity to host calls in spectrotemporal features [50]. So, cuckoo species may vary in constraints on begging call development. Some species, most likely the more generalist parasite species, show directional modifications in spectrotemporal features of begging calls by operant reinforcement learning, resulting in calls similar to those of the host species. Other species may lack this call flexibility. At the moment too little is known about the link between call plasticity and being a generalized parasite to conclude whether learning-based call flexibility should be seen as a derived or as the ancestral state in cuckoos.

### (f) Hummingbirds

**Figure 5.**
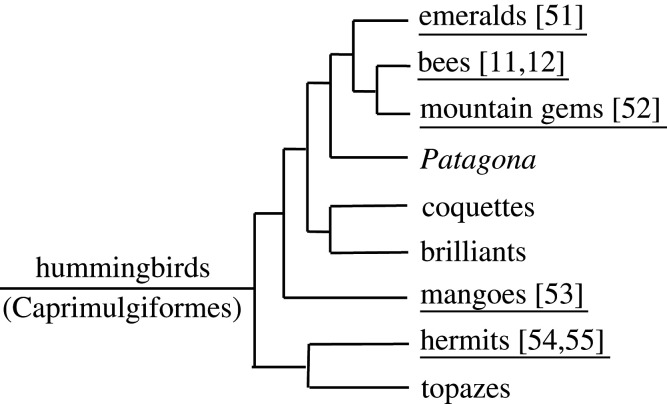
Hummingbird phylogeny, based on McGuire [56]. Underlined are those groups in which vocal learning is demonstrated or indicated from field studies. No information is available for the other groups. Numbers in brackets indicate relevant references.

Hummingbirds (Trochilidae), of which there are 365 species [20], are now included with the nightjars and their relatives in the order Caprimulgiformes. This is the third major avian order for which vocal learning is well documented. Several of the nine principal clades (figure 5) contain species for which the presence of vocal learning has been suggested, based on field observations of geographical variation in songs. In several species in which males gather at ‘leks’—specific localities to attract females—the songs are shared among males at the same lek, but differ between leks, even at a relatively short distance (e.g. [51,53]). One such species is the wedge-tailed sabrewing (belonging to the emeralds). Their songs are composed of syllables that are variable and complex in structure. Neighbouring males within a lek form ‘song neighbourhoods’ and more syllables are shared within neighbourhoods and leks than between leks [51,57,58]. Analysing this song variation in relation to genetic differentiation showed that the two are uncoupled [58]. This is also known from other lekking species, such as little and long-billed hermits [54,55], belonging to the hermit clade. Long-billed hermits sing a single song that can vary considerably between leks and is less variable within leks [55]. In addition, over time adult individuals may replace a crystallized song with a different one [55,59]. Such observations are compelling evidence for song learning. Figure 3 also shows which other clades contain species showing intra-specific song variability and song sharing among neighbours. Assuming that such song variability is an indication of the presence of vocal learning, and keeping in mind that it is only a handful of species for which such evidence has been documented (for some clades song variation is unknown), it suggests that vocal learning is ancestral in hummingbirds.

While the evidence for song learning is clear, less is known about the nature of the learning process or processes. The song changes in long-billed hermits [59] suggest the ability to memorize songs and to model the own song accordingly, analogous to many songbirds. However, how and when the initial songs develop is unclear from such observations and requires controlled experiments. Currently, such experiments are available for two hummingbird species only: the Anna's hummingbird (*Calypte anna*) and its sister species the Costa's hummingbird (*Calypte costae*; belonging to the bees). Field studies [11] on the Anna's hummingbird showed that the songs of different individuals may differ in syllable structure and song syntax. Baptista & Schuchmann [11] examined song development in four laboratory-raised individuals. One male, raised in isolation, produced a song consisting of highly variable syllables, which also lacked the usual grouping into phrases. Three other males, raised together, shared syllable types and syntax. For all three, this song deviated strongly from the natural songs of the species. These findings indicate that juveniles need a song model to develop normal songs. Recently, Johnson & Clark [12] examined song learning in the Costa's hummingbird. In contrast with the songs of Anna's hummingbird, the songs of the Costa's hummingbird have a stereotyped and universal structure consisting of a single complex syllable [60]. However, six males raised in pairs with no adequate model developed highly abnormal songs that were very similar between the members of a pair. Another 10 birds were raised singly with regular exposure to a live conspecific male while either a species-typical or a deviant sound (backward Costa's song, partial song, Costa's dive sound, Anna's hummingbird song) was played. At around 125 days of age, their songs crystallized after a subsong and plastic song phase. Two males exposed to normal song copied this song (although with some deviation in one bird), the other sounds were copied to a smaller or larger extent, with two birds exposed to the Anna song developing isolate song, suggesting constraints on what the birds considered a suitable model to copy. Follow-up experiments [61] showed that birds raised in these experiments converged in their songs with other individuals when put in aviaries later on. This showed that song learning is not limited to a sensitive phase but may also occur when adult, similar to what has been observed in other hummingbird species in the field.

The data on the Anna's and Costa's hummingbirds show clear similarities in song development. Being sister species this may be expected, although it is remarkable that the two species differ substantially in their adult song variability. Further studies are needed to understand whether the learning process in these species is representative of other hummingbird clades. In any case, the learning process shows similarities to the song learning process in songbirds by requiring exposure to a model song, song memorization at an early age, constraints on learning and the importance of social interactions.

### (g) Shorebirds

**Figure 6.**
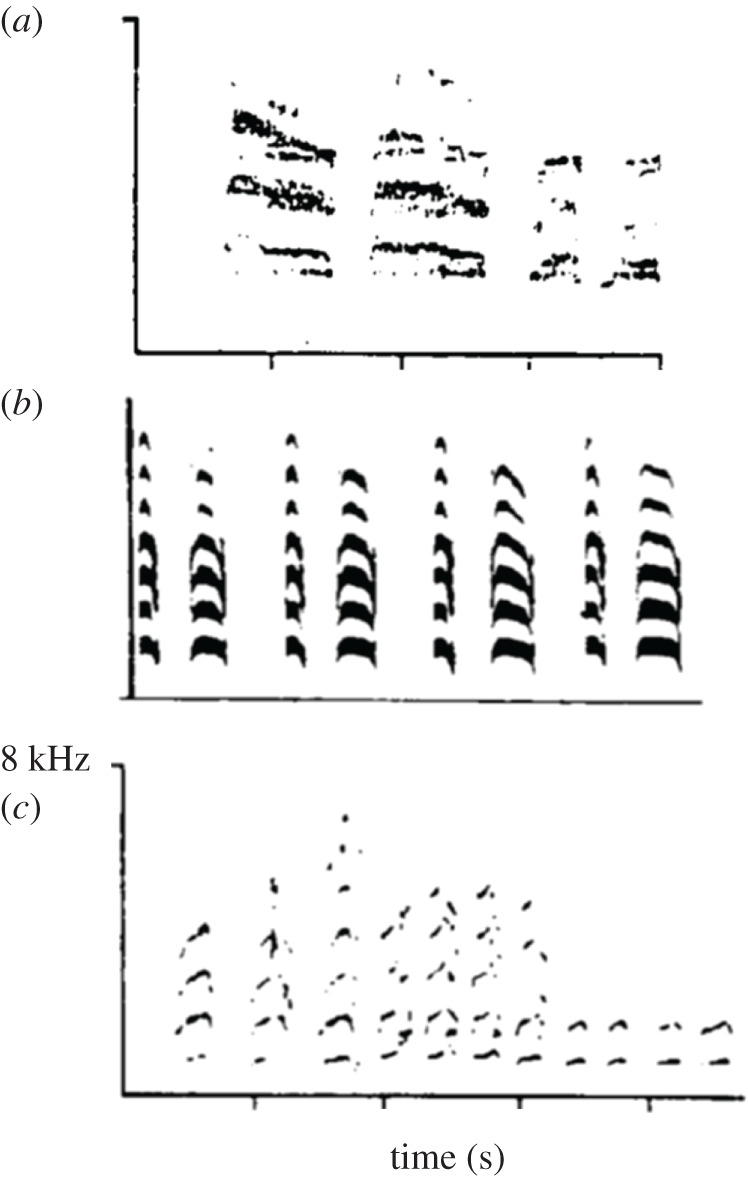
Long calls of a black-headed gull (*a*), a little gull (*b*) and a black-headed gull raised with little gulls (*c*) (from Groothuis [63] with permission from the *Netherlands Journal of Zoology*).

Gulls (Laridae—101 species [20]) are a family within the shorebirds (Charadriiformes). In a study on the development of social displays of black-headed gulls, Groothuis [62,63] reared black-headed gulls (*Larus ridibundus*) in visual isolation, in small groups or with agemates of other gull species. Among the birds reared in isolation or in small groups of conspecifics, several showed pronounced and persistent deviations in call development [62]. Also, one bird out of four that were individually reared with little gulls (*Larus minutus*) produced a stereotyped long call strongly deviating from the normal temporal structure and harsh quality of black-headed gull long calls. Instead, it showed a rapid succession of short notes and a harmonic structure characteristic of little gull long calls [63] (figure 6). Although the call thus showed a clear change towards little gull calls, it was not identical to the long calls of little gulls and still possessed a decline in pitch over successive call notes as present in black-headed gulls. Groothuis [63] ascribed the deviations to the intense social interactions within the small groups in which the reactions of other individuals may reinforce particular call and display deviations. The deviating calls of isolated reared birds and the convergence on little gull long calls in one bird may indicate a role of learning in vocal development and call for further exploration of vocal learning in this and other species of this clade.

### (h) Landfowl

Konishi's study [13], in which deafened chickens developed normal species-specific vocalizations, indicated the absence of vocal learning in this member of the order Galliformes (388 species—[20]). However, Sparling [64] described observations suggesting that adult greater prairie chickens (*Tympanuchus cupido*) and sharp-tailed grouse (*Pedioecetes phasianellus*) adjusted some aspects of their vocalizations to those heard from other individuals, which he interpreted as indicating vocal learning. Male prairie chickens displaying on leks on which there were F1 hybrids of prairie chickens and sharp-tailed grouse, started to produce a three-note call that was originally produced by F1 hybrids only. A caveat in these observations is that hybrid vocalizations can be very variable and that there is also intra-individual variation in those of the parental species (J Augustin 2019, personal communication). So, it can be hard to assess whether a deviating call, presumed to originate from learning, really is a newly learned one or a rarely used existing variant. Another sharp-tailed grouse was reported to modify the note interval and note duration towards a playback of an artificially changed call sequence [64]. As there is no mention of persistence of the vocal modification after the exposure, this convergence might also result from vocal accommodation, as described for some parrots. Therefore, while some form of vocal learning cannot be excluded, controlled experiments are needed to examine its presence among Galliformes.

### (i) Waterfowl

**Figure 7.**
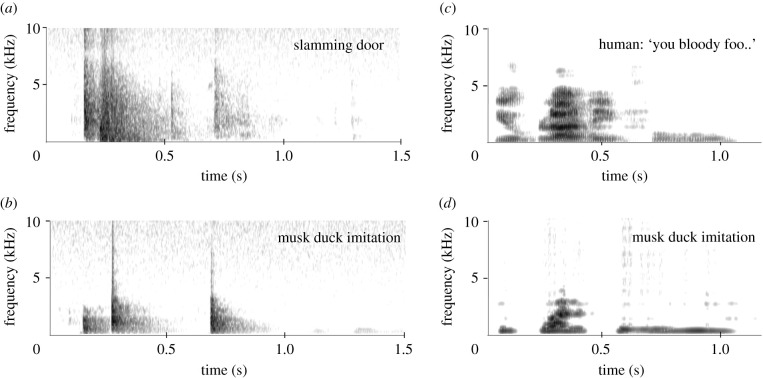
Imitations (*b*,*d*) by the Australian musk duck ‘Ripper’ of the opening and closing of a metal door (*a*) and a human voice (not the one to which the duck had been exposed) (*c*).

Several of the 176 species [20] of Anseriformes have been domesticated and the behaviour and its development in many species has been studied extensively, both in the field and in captivity. This has not resulted in any report suggesting vocal learning in this clade, with one notable exception: the Australian musk duck, for which vocal imitations have been documented (e.g. [65]). These imitations are present in recordings of one hand-reared individual, Ripper, and also in those of a second captive-reared duck. They are described in more detail in another paper in this issue [66]. Ripper's imitations consist of the imitation of a slamming metal door (figure 7*a*,*b*), resembling the sound of a door next to the place where the duck was raised, and of speech-like sounds. One of these can be interpreted as ‘you bloody foo(l/d)’ (figure 7*c*,*d*), a phrase most likely originating from a carer of the bird. The second duck copied quacks of a Pacific black duck. All copied sounds are produced during a visual display, partly instead of the loud whistle sound normally accompanying this display. In addition, field recordings of the species in different parts of its range show the geographic variation of the vocalizations accompanying the display [65,67]. Also, individuals raised in captivity commonly show deviations in their vocalizations [65]. The difference from the normal species-specific vocalizations and the memorization of auditory models during early development while producing them later on resemble the sensory-motor learning observed in songbirds, rather than the modification of an existing vocalization into a different one by conditioning. So, musk duck clearly demonstrates vocal production learning.

## 2. Discussion

The above overview shows that the vocal characteristics in a range of species can be affected by experience. The extent of the modifications vary from limited or temporary adjustments, such as modifying an existing vocalization to become more similar or dissimilar from a vocalization of a particular conspecific, to producing long-lasting strongly deviating sounds copied from other species or from non-natural sources. Table 1 presents an overview of the findings. Below, I first discuss the distribution of experience-based vocal modifications among birds. Next, I reflect on the types of learning that may be present in the various groups and the implications for future research.

**Table 1.** Overview of vocal modifications and mechanisms among bird orders (√, presence; √?, possible presence; ?, presence is unclear; empty box, lack of evidence or knowledge).

	type of vocal modification						mechanism		
			modification of existing vocalization			novel vocalization			
avian order	towards conspecific model	away from conspecific model	towards allospecific model	conspecific model	allospecific/other sound model	no model^a^	template based	reinforcement based	other^b^
oscine songbirds	√	?	√?	√	√	√	√	√	√?
suboscines	√			√	√	√	√	√?	
parrots	√	√	√	√	√	√	√	√	√
woodpeckers and allies					?				
loons		√					√?	√?	
cuckoos			√					√	
hummingbirds	√?			√	√?	√	√	√?	
shorebirds					√	√	?	√?	
landfowl	?						?		?
waterfowl				√?	√	√	√	?	

^a^Birds raised without a model, e.g. in isolation, develop vocalizations deviating substantially from normal species-specific vocalizations. ^b^Vocal changes likely to originate from some other process than template-based or reinforcement-based learning. This applies in particular to rapid vocal convergence or divergence during interactions, for which it is unclear how they might arise and whether they are transient or become memorized.

With respect to the distribution of vocal learning, convincing evidence for some type of vocal modification is present for species belonging to several avian orders (table 1)—apart from oscine songbirds, parrots and hummingbirds these are suboscines, cuckoos, a gull, a duck and a loon. For landfowl, the evidence is ambiguous, while an earlier report on vocal imitation in a toucannet may have been premature. The various examples of vocal learning are scattered over the avian phylogenetic tree, including one of its earliest diverging branches: the waterfowl. This suggests a widespread potential for the evolution of some form of vocal learning. Nevertheless, one may argue that the limited number of species showing evidence of vocal learning still indicates it is a rare phenomenon. This may be true. On the other hand, many taxa have never been examined for the presence of any form of vocal learning and several of the above-mentioned findings were coincidental. The learning only became apparent in exceptional circumstances in which normal development was disrupted or redirected. Hence, although it is too far-fetched to see such examples as the tip of an iceberg, it may indicate that systematic studies of cross-fostered and hand-raised isolated birds might reveal more examples of vocal learning. Also, modern techniques allow more sophisticated and quantitative analyses of vocalizations, which might lead to the discovery of more subtle examples of vocal convergence or divergence such as observed in loons and parrots. A search for further examples of vocal learning might focus on species in which we find vocalizations consisting of sequences of elements varying in several spectral and temporal features, for instance some waders, hornbills, barbets, fruit doves and penguins. Such more complex vocalizations may indicate the presence of a more complex vocal control, which might also show some flexibility. In addition, species characterized by intense and prolonged social interactions between parents and offspring or with social partners and group mates might be more likely to have vocalizations open to modifications. Such social interactions with a live model are a very prominent factor affecting vocal learning in several songbirds and parrots and the allospecific copying in bellbirds, the musk duck and the black-headed gull also arose from intense social interactions with another species or a carer. This is a striking parallel with some of the findings in mammals, in which vocal learning was unexpectedly demonstrated in some isolated individuals raised in close interaction with carers such as the seal Hoover [5] and the Asian elephant Koshik [6]. Additionally, a search for vocal learning may benefit from sampling different types of vocalizations as in all taxa showing vocal learning, including songbirds and parrots, experience affects some but not all vocalizations. It may also not only be complex vocalizations that are affected by learning, but also, as observed in songbirds [68], seemingly simple ones, such as calls. Finally, the physical and physiological characteristics of the vocal organ, the syrinx, might constrain the parameters which indicate the presence of vocal learning. The location, structure and complexity of the syrinx vary greatly among different species (e.g. [69,70]). Birds with more complex structures and musculature might therefore be capable of producing a larger variety of sounds. We thus cannot exclude the possibility that in some cases in which a species shows only partial imitations of sounds of another species or of another source this results from a constraint of the vocal organ rather than the neural mechanism—the bird may not be able to fully match what it has learned. Such constraints might affect some vocal parameters more than others [16]. For instance, the duration of vocal units and pauses between them may result from putting the syrinx in a vocalizing position, while frequency modulations may require more complex actions and coordination of syringeal muscles. Temporal patterns may therefore seem to be more susceptible to the impact of experience than frequency modulations but this difference may originate from constraints at the periphery rather than at the central neural level.

What the overview also demonstrates is that vocal production learning is not a single unidimensional process (table 1). Some examples, such as the convergence in call characteristics during an interaction as observed in some parrot species, might be considered examples of vocal accommodation if the convergence leaves no trace after the vocal exchange. However, most examples discussed above concern longer lasting directional modifications of vocalizations or the emergence of new ones. These modifications vary in their degree of change, an observation that has led to the model of a continuum or a graded scale of learning [71], with stronger constraints on learning in some species compared to others. This model may suggest that the same type of learning is involved in all these cases, showing differences in the degree of expression. The overview above indicates that there are several learning mechanisms. One of these is the sensorimotor learning exemplified in songbirds in which a template is formed based on hearing a particular vocalization before or coinciding with the start of vocal production and serving as the basis for shaping the individual's own vocalizations. Such template-based learning is most likely also present in parrots, hummingbirds, the suboscine bellbirds and the musk duck. In these groups, it most likely evolved independently and it still remains to be seen how similar these groups are in the details of the process. However, vocal modifications may also originate from the second type of learning, most clearly represented in the Horsfield's bronze cuckoo and in budgerigars. Here, an initially rather variable vocalization becomes modified by differential responses that the bird receives upon producing different vocal variants. In this case, clear similarities between this sound and an identifiable model sound cannot be based on a previously acquired template. Such reinforcement-based modification of vocalizations is also shown by some songbirds, such as cowbirds [72] and zebra finches [73], and deserves more extensive study, as it may underlie vocal modifications in parrots, loons, cuckoos and gulls and be present more broadly in other groups. Social reinforcers seem the main driver of these modifications, although other rewards such as food may also result in changes. A possible third process might be that of adjusting an already existing vocalization to one resembling that of other individuals, as occurs in several parrot species in response to playback within a limited time frame. The difference from sensorimotor learning is that there is already a pre-existing vocalization, which is subsequently adjusted and modified. The difference from reinforcement learning is that it is vocal input rather than selective reinforcement giving rise to the changes. The nature of this process is currently still rather obscure, as is its relation to a process like vocal accommodation. So, at the moment, much is still unclear about the details of the different processes as they occur in various groups, as well as about how these processes relate to each other. As mentioned for parrots, it may well be that some of these processes act in cooperation, as also occurs in humans where young infants do not learn from exposure only, but also from the responses of others to their utterances.

To conclude, different mechanisms can result in ‘vocal learning’, and each of these is present to a larger or lesser degree in distinct branches of the avian phylogeny. This indicates that the presence of vocal learning is the result of convergence from a series of independent evolutionary trajectories originating from different starting points. The starting point for vocal learning due to selective reinforcement, for instance, might be operant conditioning more generally. This is assumed to be an evolutionarily ancient type of learning process, and, as with other types of motor output, the vocal motor output might also have come under the control of reinforcers. Vocal accommodation may have been the basis of the learning process that nowadays induces longer lasting call convergence or divergence. The ability for vocal convergence might in turn have given rise to more extensive forms of vocal imitation. Along with variation in the mechanisms at the behavioural level, variation should be expected at the neural level. Even the same type of learning, as identifiable by its behavioural characteristics, e.g. ‘template-based learning’, may vary in details if it has arisen independently in different taxa. For the vocal learning in the Australian musk duck, any knowledge on the neural substrate involved is lacking. And although comparative studies have shown some deep homologies in the neural circuits and gene network underlying vocal learning in songbirds, hummingbirds and parrots [74], these circuits also show differences for which it is not yet known how they relate to differences in the learning processes involved. Also, similar to behavioural mechanisms, different parts of the neural systems in different taxa may have converged to do the same job.

Thus, the diversity in vocal learning observed among birds, as revealed in this review, emphasizes that we still have only a fragmentary knowledge of its distribution, the processes involved, their underlying neural substrates and circuits, and how these processes may have evolved. Nevertheless, I hope to have demonstrated that broadening the scope of research beyond songbirds, parrots and hummingbirds and applying a more fine-tuned analysis of the nature of vocal learning in different groups is likely to be very profitable for understanding how vocal learning can be brought about and how it might have evolved. Examining this variation will also increase the value of comparative research for understanding vocal learning in humans.

## Data accessibility

This article has no additional data.
